# Elevated Systemic IL-6 Levels in Patients with Aneurysmal Subarachnoid Hemorrhage Is an Unspecific Marker for Post-SAH Complications

**DOI:** 10.3390/ijms18122580

**Published:** 2017-12-01

**Authors:** Shafqat Rasul Chaudhry, Birgit Stoffel-Wagner, Thomas Mehari Kinfe, Erdem Güresir, Hartmut Vatter, Dirk Dietrich, Alf Lamprecht, Sajjad Muhammad

**Affiliations:** 1 Department of Neurosurgery, University Hospital Bonn, D-53127 Bonn, Germany; shafqat.chaudhry@ukb.uni-bonn.de (S.R.C.); Thomas.kinfe@ukb.uni-bonn.de (T.M.K.); erdem.gueresir@ukb.uni-bonn.de (E.G.); Hartmut.Vatter@ukbonn.de (H.V.); dirk.dietrich@uni-bonn.de (D.D.); 2 Department of Pharmaceutics, University of Bonn, D-53121 Bonn, Germany; alf.lamprecht@uni-bonn.de; 3 Department of Clinical Chemistry and Clinical Pharmacology, University Hospital Bonn, D-53127 Bonn, Germany; birgit.stoffel-wagner@ukb.uni-bonn.de

**Keywords:** aneurysm, subarachnoid hemorrhage, inflammation, interleukin-6 (IL-6), clinical outcome, post-SAH complications

## Abstract

**Background:** Aneurysmal subarachnoid hemorrhage (aSAH) is still a fatal and morbid disease, although bleeding aneurysms can be secured in almost all cases. Occurrence of post-SAH complications including cerebral vasospasm, delayed cerebral ischemia, hydrocephalus, epilepsy, and infections are the main determinants of clinical outcome. Hence, it is important to search for early predictors for specific post-SAH complications to treat these complications properly. Both cellular and molecular (cytokines) inflammation play a key role after aSAH during the phase of occurrence of post-SAH complications. Interleukin-6 (IL-6) is a well-known cytokine that has been extensively analyzed in cerebrospinal fluid (CSF) of patients after aSAH, but detailed studies exploring the role of systemic IL-6 in aSAH associated complications and its impact on early clinical outcome prediction are lacking. The current study aims to analyze the systemic IL-6 levels over two weeks after bleeding and its role in post-SAH complications. **Methods:** We recruited 80 aSAH patients prospectively who underwent peripheral venous blood withdrawal in serum gel tubes. The blood was centrifuged to harvest the serum, which was immediately frozen at −80 °C until analysis. Serum IL-6 levels were quantified using Immulite immunoassay system. Patient records including age, gender, post-SAH complications, aneurysm treatment, and clinical outcome (modified Rankin scale and Glasgow outcome scale) were retrieved to allow different subgroup analysis. **Results:** Serum IL-6 levels were significantly raised after aSAH compared to healthy controls over the first two weeks after hemorrhage. Serum IL-6 levels were found to be significantly elevated in aSAH patients presenting with higher Hunt and Hess grades, increasing age, and both intraventricular and intracerebral hemorrhage. Interestingly, serum IL-6 was also significantly raised in aSAH patients who developed seizures, cerebral vasospasm (CVS), and chronic hydrocephalus. IL-6 levels were sensitive to the development of infections and showed an increase in patients who developed pneumoniae. Intriguingly, we found a delayed increase in serum IL-6 in patients developing cerebral infarction. Finally, IL-6 levels were significantly higher in patients presenting with poor clinical outcome in comparison to good clinical outcome at discharge from hospital. **Conclusion:** Serum IL-6 levels were elevated early after aSAH and remained high over the two weeks after initial bleeding. Serum IL-6 was elevated in different aSAH associated complications, acting as a non-specific marker for post-SAH complications and an important biomarker for clinical outcome at discharge.

## 1. Introduction

Aneurysmal subarachnoid hemorrhage (aSAH) is a rare form of stroke accounting for about 2–5% of cases of all strokes and has a very high mortality rate reaching above 40% [1]. The affected patients are much younger than the patients with ischemic stroke, and this poses even more economic burden on the society [2,3]. The morbidity and mortality after aSAH is still very high, although bleeding aneurysms can be secured in almost all cases either by endovascular coiling or with micro-neurosurgical clipping [4]. The reason for poor clinical outcome after aSAH is the occurrence of multiple post-hemorrhagic complications including cerebral vasospasm (CVS), delayed cerebral ischemia (DCI), hydrocephalus, symptomatic epilepsy, and systemic infections [5,6,7,8,9,10]. Delayed cerebral ischemia, a multi-factorial phenomenon, but mainly secondary to CVS, is the main player in the prediction of clinical outcome [11]. Proper treatment and management of post-SAH complications may improve the clinical outcome. However, the current options to treat CVS can themselves cause cardiovascular complications [12]. Hence, it is important to search for early predictors of DCI and other post-SAH complications to treat them timely and properly. Both cellular and molecular (cytokines) inflammation play a key role during the occurrence of post-SAH complications. Inflammatory conditions are known to be associated with development of delayed cerebral ischemia and poor functional outcome [11,13]. Pharmacological means to antagonize the pro-inflammatory molecules have been shown to reduce CVS and confer neuroprotection [14].

Interleukin-6 (IL-6) is a pleiotropic cytokine with hormone like activity that can influence vascular and metabolic diseases [15,16,17]. IL-6 signals via IL-6R (CD126, type 1 cytokine α-receptor subunit) and gp130 (CD130, β-receptor subunit) and involves downstream signaling pathways such as GTPase Ras-Raf, Mitogen activated protein kinases (MAPK) and Janus kinase-Signal transducer and activator of transcription (JAK-STAT) [18,19]. Three modes of IL-6 signaling have been identified: classical, involving membrane bound IL-6R and gp130; trans-signaling, dependent on soluble IL-6R whereby only gp130 expressing cells can gain response to the IL-6-sIL-6R complex, and recently identified cluster signaling in which dendritic cells harboring the IL-6-IL-6R complex in their membranes engage gp130 on the target cell membranes [18,19,20]. Depending on the context of disease, IL-6 has both pro-inflammatory and anti-inflammatory effects [18]. In acute pathological conditions, including aSAH, IL-6 stimulates the neuroinflammatory response [21] that may contribute to the disease progression. IL-6 levels in cerebrospinal fluid (CSF) of patients after aSAH have been shown to be associated with occurrence of cerebral vasospasm and clinical outcome [22,23,24,25,26,27,28]. Moreover, elevated early serum IL-6 levels predict an unfavorable clinical outcome [29,30,31,32]. However, detailed studies exploring the kinetics of IL-6 release in systemic circulation over the two weeks after aSAH and its association with post-SAH complications and its impact on early clinical outcome are lacking. The current study was aimed at analyzing the systemic IL-6 levels over two weeks (covering the peak time to develop the post-SAH complications) after bleeding and its role in post-SAH complications.

## 2. Results

### 2.1. Patient Characteristics

The mean age of the aSAH patients recruited in our study was 56.97 ± 12.00 (mean ± SD) with a predominance of female patients (62.5%). Interestingly, the median H&H score, Fischer score, GOS score, and mRS score of our patient population was three. The detailed characteristics of these aSAH patients are represented in Table 1.

### 2.2. Elevated Systemic IL-6 after Aneurysmal Subarachnoid Hemorrhage (aSAH)

**Figure 1:**
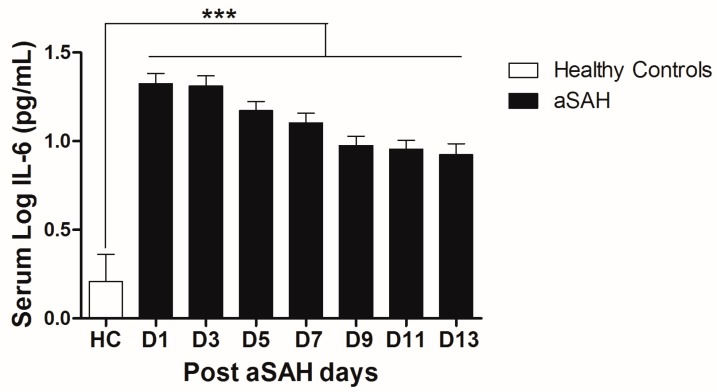
Comparison of serum IL-6 levels among healthy controls and aneurysmal subarachnoid hemorrhage (aSAH) patients. HC = Healthy controls, *n* = 10, aSAH = Aneurysmal subarachnoid hemorrhage, *n* = 80, D1–D13 = Day 1–Day 13, Unpaired *t* test, *p* < 0.001 (***), error bars represent SEM.

**Figure 2:**
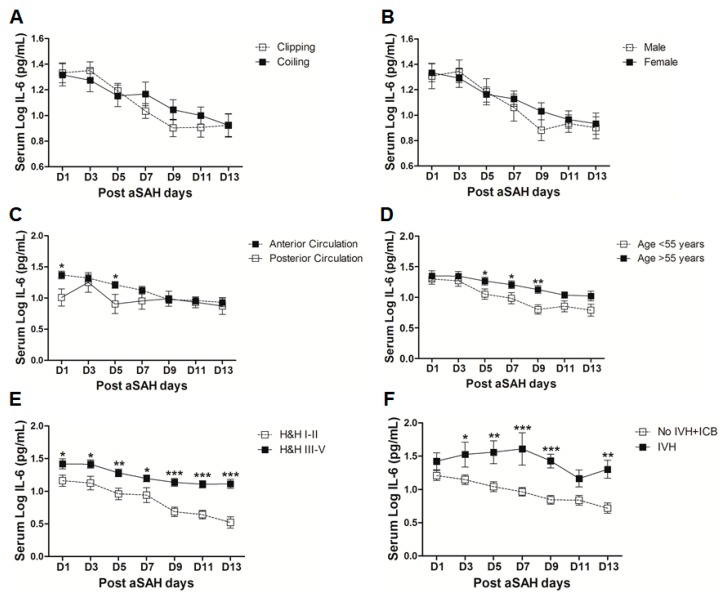
Comparison of serum interleukin-6 (IL-6) levels among aSAH patients with different characteristics. (**A**) Comparison of serum IL-6 levels in aSAH patients undergoing repair of aneurysm by neurosurgical clipping (*n* = 39) or endovascular coiling (*n* = 41); (**B**) Comparison of serum IL-6 levels in male (*n* = 30) and female (*n* = 50) aSAH patients; (**C**) Comparison of serum IL-6 levels among aSAH patients with anterior circulation aneurysms (*n* = 69) and posterior circulation aneurysms (*n* = 11); (**D**) Comparison of serum IL-6 levels among aSAH patients below (*n* = 37) and above 55 years of age (*n* = 43); (**E**) Comparison of serum IL-6 levels among aSAH patients with good Hunt and Hess (H&H) grades (*n* = 29) and poor H&H grades (*n* = 51); (**F**) Comparison of serum IL-6 levels among aSAH patients showing no intraventricular hemorrhage and intracerebral bleeding (IVH + ICB) (*n* = 43) and IVH (*n* = 10). Unpaired *t* test to compare the two groups, *p* < 0.05 (*), *p* < 0.01 (**), *p* < 0.001 (***), error bars represent SEM.

**Figure 3:**
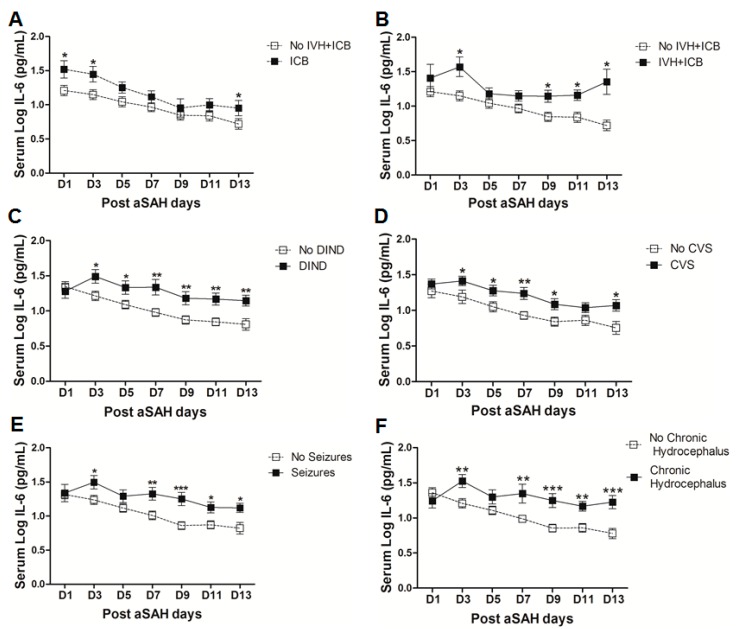
(**A**) Comparison of serum IL-6 levels among aSAH patients showing no IVH + ICB (*n* = 43) and ICB (*n* = 16). (**B**) Comparison of serum IL-6 levels among aSAH patients showing no IVH + ICB (*n* = 43) and both IVH + ICB (*n* = 11). (**C**) Comparison of serum IL-6 in patients displaying delayed ischemic neurological deficits (DIND) (*n* = 28) and no DIND (*n* = 52). (**D**) Comparison of serum IL-6 in aSAH patients who developed cerebral vasospasm (CVS) (*n* = 44) and no CVS (*n* = 36). (**E**) Comparison of serum IL-6 in aSAH patients who developed seizures (*n* = 24) and no seizures (*n* = 56). (**F**) Comparison of serum IL-6 in aSAH patients who developed chronic hydrocephalus (*n* = 25) and no chronic hydrocephalus (*n* = 55). Unpaired *t* test to compare the two groups, *p* < 0.05 (*), *p* < 0.01 (**), *p* < 0.001 (***), error bars represent SEM.

IL-6 is a well-known pro-inflammatory cytokine that is secreted early after aSAH [22,23]. To analyze the systemic IL-6 release starting from day of ictus over the time of development of post-SAH complications, we determined the serum IL-6 levels in aSAH patients and healthy controls. Serum IL-6 levels were significantly raised in systemic circulation after aSAH as compared to that of healthy controls and remained persistently high till day 13 after bleeding (Figure 1). For further analysis, the data was dichotomized into two groups. Serum IL-6 levels remained significantly raised in patients presenting with poor H&H grade, reflecting the inflammatory response as severity of aSAH (Figure 2E). There was no significant difference in IL-6 levels among patients undergoing neurosurgical clipping or endovascular coiling (Figure 2A) and the same was true for gender comparison (Figure 2B), reflecting the fact that gender and craniotomy do not alter the systemic IL-6 levels. Serum IL-6 levels, only at day 1 and day 5, were significantly higher in patients having anterior circulation aneurysms (Figure 2C). Most of the studies describe mean age of aSAH patients around 55 years. So, we dichotomized patients into two groups. The first group above the age of 55 years and the second below the age of 55 years, and we found that serum IL-6 levels tend to be higher in patients with age above 55 years. This tendency reached to a significant difference at days 5–7 (Figure 2D), reflecting the possibility of more severe systemic inflammation at higher age. Serum IL-6 levels were significantly raised in aSAH patients who presented with additional intraventricular or intracerebral bleeding or both simultaneously at several days in comparison to that of patients who had only blood in subarachnoid space (Figure 2F and Figure 3A,B).

### 2.3. Serum IL-6 in Post-SAH Complications

To analyze the influence of systemic IL-6 levels on the occurrence of post-SAH complications, we dichotomized the patients into two groups, one who developed the complications and another without those complications. The serum IL-6 levels were then compared in both groups. Serum IL-6 levels were found to be significantly raised after aSAH in patients who displayed delayed ischemic neurological deficits (DIND) throughout the assessment days (Figure 3C). Serum IL-6 levels were significantly associated with DIND at all days except at day 1 (OR = 6.3, *p* = 0.01, 95% CI = 1.5–26.0 at day 3; OR = 4.6, *p* = 0.04, 95% CI = 1.1–20.2 at day 5; OR = 9.4, *p* = 0.01, 95% CI = 1.7–52.7 at day 7; OR = 13.1, *p* = 0.005, 95% CI = 2.2–79.4 at day 9; OR = 30.5, *p* = 0.01, 95% CI = 2.6–356.7 at day 11; OR = 7.8, *p* = 0.01, 95% CI = 1.5–40.0 at day 13) even after controlling for age, gender, H&H score, Fischer score, IVH, ICB, aneurysm location, and treatment (see also supplementary Table S1). Similarly, patients who were diagnosed with angiographic vasospasm with significant perfusion deficits had significantly elevated serum IL-6 levels (Figure 3D). Multivariate analysis also showed significant association of serum IL-6 with CVS at day 3 (OR = 4.6, *p* = 0.025, 95% CI = 1.2–17.1), day 7 (OR = 16, *p* = 0.012, 95% CI = 1.9–137.4), day 9 (OR = 8.6, *p* = 0.014, 95% CI = 1.5–47.7), and day13 (OR = 5.8, *p* = 0.031, 95% CI = 1.2–28.4) after adjusting for confounders (see also supplementary Table S1). These findings reflect the involvement of systemic inflammation in the development of CVS or DIND that is consistent with the previous findings [29,33,34]. Pharmacological interventions to interrupt IL-6 signaling reversed vasospasm in a rat femoral artery vasospasm model [14,35]. Interestingly, soluble gp130 antagonizing IL-6 signaling has been described to follow a parallel increase with IL-6 on day 1 after aSAH in CSF and then gradually declines, relieving IL-6 from regulatory check and paving the way to the development of vasospasm [36]. Except at day 1 and day 5, serum IL-6 levels were significantly increased in patients who developed seizures as compared to IL-6 levels in those who did not (Figure 3E), and seizures have been already known to be associated with increased risk of rebleeding [10]. However, serum IL-6 levels were independently associated with seizures only at day 7 (OR = 4.2, *p* = 0.03, 95% CI = 1.1–15.6) and day 9 (OR = 9.5, *p* = 0.02, 95% CI = 1.4–63.8) (see also Table S1). Similarly, IL-6 levels were upregulated in patients who developed chronic hydrocephalus and required ventriculoperitoneal shunt placement (Figure 3F). After controlling for the effects of confounders, serum IL-6 levels were significantly associated with chronic hydrocephalus at day 9 (OR = 8.1, *p* = 0.025, 95% CI = 1.3–50.1) and day 13 (OR = 9.6, *p* = 0.025, 95% CI = 1.3–69.3) (see also Table S1).

**Figure 4:**
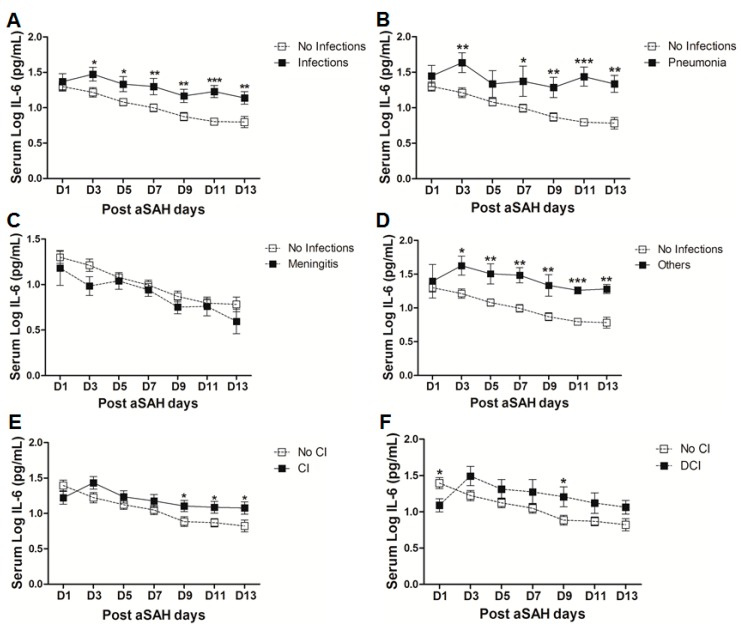
(**A**) Comparison of serum IL-6 levels in aSAH patients with infections (*n* = 30) and without infections (50). (**B**) Comparison of serum IL-6 levels in aSAH patients presenting with only pneumonia (*n* = 14) and no infections (*n* = 50). (**C**) Comparison of serum IL-6 levels in aSAH patients presenting with only meningitis (*n* = 7) and no infections (*n* = 50). (**D**) Comparison of serum IL-6 levels in aSAH patients presenting with other infections (*n* = 9) and no infections (*n* = 50). (**E**) Comparison of serum IL-6 levels among patients with cerebral ischemia (CI) (*n* = 33) and no CI (*n* = 47). (**F**) Comparison of serum IL-6 levels in patients with delayed cerebral ischemia (DCI) (*n* = 16) and no CI (*n* = 47). Unpaired *t* test to compare the two groups, *p* < 0.05 (*), *p* < 0.01 (**), *p* < 0.001 (***), error bars represent SEM.

A significant proportion of aSAH patients suffer from nosocomial infections after the initial bleeding, showing the possibility of immunosuppression after aSAH [5,37]. We found that serum IL-6 levels were significantly raised in aSAH patients who contracted infections (Figure 4A). However, controlling for other factors mentioned above, serum IL-6 levels showed association with infections at day 11 (OR = 116, *p* = 0.01, 95% CI = 4.0–3395.2) and at day 13 (OR = 11.9, *p* = 0.03, 95% CI = 1.2–117.6) (see also supplementary Table S1). Further dissection of the data into sub-groups of patients who developed pneumonia, meningitis, and other infections (having other infections such as urinary tract infections (UTI) or in addition to pneumonia or meningitis) revealed distinct differences in serum IL-6 levels among these sub-groups. Interestingly, aSAH patients contracting pneumonia or other infections had significantly higher serum IL-6 levels as compared to those of non-infectious aSAH patients (Figure 4B,D). Multivariate analysis only showed significant association of serum IL-6 with pneumonia at days 9, 11, 13 (OR = 31.1, *p* = 0.019, 95% CI = 1.8–548.3 at day 9; OR = 1548.8, *p* = 0.019, 95% CI = 1.5–1570483 at day 11; and OR = 45.4, *p* = 0.016, 95% CI = 2.0–1021.8 at day 13) and no association with other infections (see also Table S1). Pneumonia is frequent among aSAH patients developing later on during the course of the disease due to immunosuppression and might be responsible for the surge of IL-6 levels, which may provide resistance against *Streptococcus pneumoniae* [25,37]. Serum IL-6 levels were unchanged in patients with meningitis as compared to aSAH patients without infections (Figure 4C) except on day 3 as shown by multivariate analysis revealing a negative independent association between serum IL-6 and meningitis (OR = 0.002, *p* = 0.024, 95% CI = 0.0–0.4) (see also Table S1). This might be explained by multiple factors including a localized inflammatory response as elicited by bacterial meningitis, immunosuppression, downregulation of IL-6 due to additional inflammatory burden posed by meningitis, and probably due to small number of patients contracting meningitis [37,38].

Finally, we evaluated serum IL-6 levels among patients who developed cerebral infarction and found a delayed significant elevation of serum IL-6 at days 9 and 13 (Figure 4E), but a negative association of serum IL-6 and cerebral ischemia existed at day 1 after controlling for other factors (OR = 0.2, *p* = 0.024, 95% CI = 0.1–0.8). Further dichotomy of patients who developed cerebral ischemia into two sub-groups including ischemia due to intervention (interventional CI) and the second group with delayed cerebral ischemia (DCI), showed that serum IL-6 levels were non-significantly raised later in the interventional CI group. However, interestingly serum IL-6 levels were significantly lower at day 1 in patients who developed DCI as compared to the patients without delayed cerebral ischemia. However, the group of patients who developed DCI showed an increase in serum IL-6 levels at day 3 that remained elevated and reached significance at day 9 (Figure 4F). Similarly, multivariate analysis showed that serum IL-6 levels were negatively associated with DCI at day 1 (OR = 0.2, *p* = 0.026, 95% CI = 0.03–0.8) and positively associated at day 3 (OR = 6.6, *p* = 0.04, 95% CI = 1.1–40.2), day 7 (OR = 8.3, *p* = 0.01, 95% CI = 1.7–39.3), and day 9 (OR = 30.4, *p* = 0.01, 95% CI = 2.3–402.1) (see also Table S1).

### 2.4. Serum IL-6 and Post-SAH Clinical Outcome

**Figure 5:**
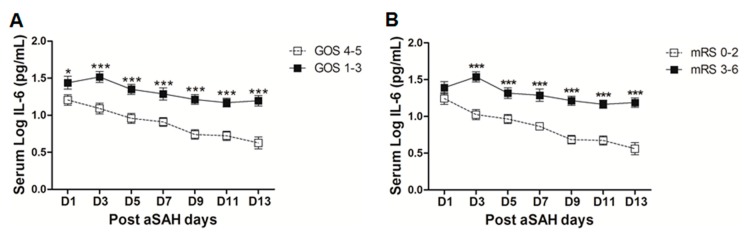
(**A**) Comparison of serum IL-6 levels among patients with good clinical outcome (GOS 4–5 = 39) and poor clinical outcome (GOS 1–3 = 41) as assessed by GOS. (**B**) Comparison of serum IL-6 levels among aSAH patients with good clinical outcome (mRS 0–2 = 35) and poor clinical outcome (mRS 3–6 = 45) as assessed by modified Rankin scale. Unpaired *t* test to compare the two groups, *p* < 0.05 (*), *p* < 0.001 (***), error bars represent SEM.

The clinical outcome of the aSAH patients at discharge was assessed by GOS and mRS. Dichotomy into good clinical outcome and poor clinical outcome by both scores revealed a significant difference in serum IL-6 levels. Serum IL-6 levels were significantly higher early at day 1 until day 13 in aSAH patients with poor clinical outcomes (GOS 1–3) as assessed by GOS (Figure 5A). After controlling for the effects of age, gender, aSAH severity, degree of bleeding, IVH, ICB, aneurysm location, and treatment, serum IL-6 levels were independently associated with poor outcome assessed by GOS at day 9 (OR = 0.1, *p* = 0.032, 95% CI = 0.01–0.8), day 11 (OR = 0.1, *p* = 0.03, 95% CI = 0.01–0.8), and day 13 (OR = 0.1, *p* = 0.02, 95% CI = 0.004–0.6) (see also Table S1). We analyzed the clinical outcome with another test battery, i.e., modified Rankin Scale (mRS). Although, there was a non-significant difference at day 1, later on a significant elevation of serum IL-6 levels in patients with poor clinical outcome (mRS 3–6) was observed as compared to that of good outcome patients (mRS 0–2, Figure 5B). In multivariate setting after controlling for above mentioned factors, serum IL-6 levels were independently associated with poor clinical outcome as assessed by mRS on all days except day 1 and day 5 (OR = 18.3, *p* = 0.019, 95% CI = 1.6–209.4 at day 3; OR = 103.6, *p* = 0.016, 95% CI = 2.4–4456.9 at day 7; OR = 311.2, *p* = 0.007, 95% CI = 4.9–19849.4 at day 9; OR = 66.8, *p* = 0.027, 95% CI = 1.6–2796.4 at day 11; and OR = 48.4, *p* = 0.01, 95% CI = 3.1–765.6 at day 13) (see also Table S1).

## 3. Discussion

Systemic IL-6 levels were elevated starting from day of bleeding and remained high over the two weeks as compared to healthy controls (Figure 1). Early brain injury is a complex row of events including elevation of intracranial pressure, reduction of cerebral blood flow, and oxidative and metabolic disturbance with acute vascular reaction leading to transient global ischemia. Elevated IL-6 levels on admission might be the response of transient global ischemia during the early brain injury. In most brain injuries including cerebral ischemia and SAH, the initial events can lead to secondary complications [39]. Circulating cytokine load plays a critical role in determining the health status of the individuals [40]. Initially released cytokines are linked to the later damage. The best example of cytokine mediated late damage is the high mobility group box 1 (HMGB1) [41,42]. We have previously shown that cytokines released from necrotic cells of an ischemic core mediate delayed brain damage in penumbra [43]. Interestingly, IL-6 signaling is known to recruit neutrophils at the site of injury in the initial phase and proteolytic processing of IL-6R on neutrophils ultimately switches the IL-6 signaling to resident cells, including cerebral vasculature cells [44]. Furthermore, IL-6 can lead to polarization of naïve T cells to Th1 or Th2 cell subpopulations [45,46]. Both subpopulations have distinct functions. Th1 type cells are pro-inflammatory in nature, but Th2 type cells have anti-inflammatory effects showing the anti-inflammatory side of IL-6.

Elevated IL-6 levels initially after aSAH may contribute to the regulation of vascular inflammation at later stages and, hence, may contribute to post-SAH complications. Occurrence of post-hemorrhagic complications is the key to determining the clinical outcome of patients after aSAH. Our data demonstrate that the patients who developed cerebral vasospasm, delayed neurological deficits, chronic hydrocephalus, and symptomatic epilepsy showed elevated systemic IL-6 levels (Figure 3C–F). Elevated serum IL-6 in most of the post-SAH complications seems to be a part of a generalized inflammatory response rather than the cause of a specific post-SAH complication. Similarly, the higher IL-6 in serum of poor grade aSAH (Figure 2E) may reflect the unspecific upregulated inflammatory response due to tissue damage. The delayed elevation of IL-6 in the serum of patients with cerebral ischemia (Figure 4E) might be secondary to ischemia; this is in line with the literature from stroke research [47]. The aSAH patients usually develop cerebral ischemia within two weeks after bleeding; the DCI is also developed late, and it has been reported that IL-6 levels tend to rise immediately before DCI [48]. In contrast to the delayed increase in serum IL-6 in CI/DCI, lower serum IL-6 levels at day 1 might be due to heterogeneity in the severity and extent of the preadmission initial intracranial pressure, transient global ischemia, and early brain injury. Furthermore, the rise of IL-6 in CSF has not been seen to be concordant to the plasma IL-6 levels and may reflect a delayed systemic inflammation as opposed to central nervous system (CNS) [25]. It may also be speculated that IL-6 levels at day 1 were lower in CI/DCI group due to a small number of patients compared to that in the no CI group. Nevertheless, elevated IL-6 levels showed association with poor clinical outcome (Figure 5), confirming previously published findings [30,31]. Our data suggest that IL-6 is not specific to predict post-SAH complications, but could be a surrogate parameter of global inflammatory burden. Interestingly, IL-6 has been regarded as the better predictor of disease activity than the most commonly used marker, C-reactive protein, because of its homeostatic basal regulation and rapid induction under different disease conditions [49,50,51]. However, in line with the existing literature, serum IL-6 levels are predictor of clinical outcome.

Our study with a human population has interesting findings, but with certain limitations. First of all, our patient population is very heterogeneous with a wide age range, inclusion of both sexes, and a diverse grade of severity of subarachnoid hemorrhage with Hunt and Hess grades I–V. All these factors may lead to an increase in the variation as reflected by our data showing that patients with age over 55 years had higher serum IL-6 levels. Even though we have controlled for such confounding factors using unconditional multivariate logistic regression, more sophisticated statistical analysis may be required. Also, the smaller sample size for subgroup analysis may have potentially undermined our findings. Hence, due to the heterogeneity of the aSAH population, the data should be interpreted carefully for any implications in the clinical setting.

Moreover, in intubated patients, the body temperature is artificially maintained during the intensive care. Elevated body temperature is known to be associated with increased IL-6 levels in systemic circulation [52]. Hence, a controlled body temperature in intubated patients may confound the systemic IL-6 levels.

Furthermore, the assessment of clinical outcome with common test batteries including GOS and mRS are not sensitive and only roughly reflect the neurological status, and, hence, the discrete changes in neurological status may be overlooked. Altogether, the complex and pleiotropic nature of IL-6 biology and its elevation in multiple post-SAH complications makes it an unspecific marker for post-SAH complications.

## 4. Methodology

### 4.1. Patient Population

We prospectively enrolled 80 aSAH patients with Hunt and Hess (H&H) grades of I–V and Fischer scores of I–IV in this study during 2013–2016. The patients presenting within 24 h of aSAH were considered for sampling. The patients with age ≤ 18 years, ischemic stroke, traumatic brain injury, onset of symptoms beyond 24 h, SAH due to arteriovenous malformations or vasculitis, pregnancy, signs of eminent death, or those who did not provide consent were excluded from the study. The aneurysm treatment either by neurosurgical clipping or endovascular coiling was based on an interdisciplinary decision. The computed tomography (CT)-angiography, CT-perfusion, or digital subtraction angiography were employed on any suspicion of cerebral vasospasm (CVS). Patients with decreased vascular diameter of more than 50% and prolonged mean transit time (MTT) above 5 s or prolonged MTT above 2 s in comparison to the contralateral side were considered to be treated. CVS was treated by induced hypertension as per institutional protocol. Prophylactic nimodipine was administered to all the patients for 21 days. Delayed ischemic neurological deficits (DIND) were considered as neurological worsening displayed by alterations in consciousness or hemiparesis or aphasia. Cerebral ischemia (CI) denoted cerebral infarction as assessed by cranial CT and was categorized into intervention related CI (interventional CI) occurring within 24 h after endovascular coiling or neurosurgical clipping and delayed cerebral ischemia (DCI). DCI represents cerebral ischemia that cannot be attributed to neurosurgical or endovascular aneurysm repair. Glasgow outcome scale (GOS) and modified Rankin scale (mRS) were used as clinical outcome measures at discharge. The study was carried out according to the guidelines of Helsinki declaration and was approved by the local ethical committee of the faculty of medicine, the University of Bonn, Germany (Reference Number: LfD 138/2011). The informed consent was obtained by the treating neurosurgeon. The patients/guardians signed the informed consent.

### 4.2. Sample Collection and Analysis

The peripheral venous blood was withdrawn into serum gel tubes (Monovette, Sarstedt, Nümbrecht, Germany) on the day of admission and on every alternate day, i.e., days 1, 3, 5, 7, 9, 11, and 13. A group of healthy volunteers served as a control with blood withdrawal at only one time-point. The blood was centrifuged at 3000 rpm in a benchtop centrifuge (Sigma, Osterode am Harz, Germany) for 10 mins to obtain serum. The serum was immediately frozen at −80 °C until analysis. Serum IL-6 levels were determined at the Department of Clinical Chemistry and Clinical Pharmacology, the University of Bonn as a part of routine diagnostics using the Immulite immunometric assay based on a solid-phase enzyme-labelled and chemiluminescence principle [30,31].

### 4.3. Statistical Analysis

Serum IL-6 levels were log normalized and were represented as mean ± SEM. Serum IL-6 levels among controls and aSAH groups (two groups) were compared using unpaired *t* test. For subgroup analysis of different aSAH patient-associated characteristics (age, gender, H&H and Fischer scores, aneurysm treatment modality, aneurysm locality, intraventricular and intracerebral bleeding), post-SAH complications (CVS, DIND, chronic hydrocephalus, seizures, infections, cerebral ischemia), and clinical outcomes (GOS, mRS), data were dichotomized into two groups and analyzed by unpaired *t* test. A *p* value less than 0.05 was considered as a significant difference. The data were analyzed using GraphPad Prism 5.00 (GraphPad Software, San Diego, CA, USA). Multivariate analysis was performed to assess the association of IL-6 with different complications and clinical outcomes after adjusting for aSAH patients’ age, gender, H&H score, Fischer score, IVH, ICB, aneurysm location, and treatment using SPSS (IBM SPSS version 24 for windows, IBM Corp., Armonk, NY, USA). The categorical variables were transformed into dichotomized dummy variables (H&H score, Fischer score, IVH, ICB, aneurysm location, aneurysm treatment, presence or absence of complications, and clinical outcome), while age and IL-6 levels were treated as continuous variables. Then, unconditional multivariate logistic regression was performed by simultaneously entering all the above mentioned predictor variables to assess the association of IL-6 levels at corresponding days with different complications and clinical outcomes while controlling for other variables in the model. The goodness of the model fit was assessed using Hosmer and Lameshow test. The detailed information about individual ORs and *p* values is presented in Table S1.

## 5. Conclusions

Serum IL-6 levels were elevated early after aSAH and remained high over the two weeks after initial bleeding. Serum IL-6 was elevated in different aSAH-associated complications, which can be used as a non-specific marker for post-SAH complications and is an important biomarker for clinical outcome at discharge.

## Figures and Tables

**Table 1:** Clinical characteristics of aneurysmal subarachnoid hemorrhage (aSAH) patients.

Number of aSAH Patients	80
Age (years) (mean ± SD)	56.97 (±12.00)
Females (%)	62.5%
Treatment modality	
Neurosurgical clipping (%)	48.8%
Endovascular coiling (%)	51.3%
Intraventricularhemorrhage: IVH (%)	12.5%
Intracerebralbleeding: ICB (%)	20.0%
ICB and IVH (%)	13.8%
Hunt and Hess grade (median)	3
1 (%)	6.3%
2 (%)	30.0%
3 (%)	28.8%
4 (%)	16.3%
5 (%)	18.8%
Fischer grade (median)	3
1 (%)	1.3%
2 (%)	2.5%
3 (%)	83.8%
4 (%)	12.5%
Cerebral vasospasm (CVS) (%)	55.0%
Cerebral ischemia (CI) (%)	41.3%
Intervention related CI (%)	21.3%
Delayed cerebral ischemia (DCI) (%)	20.0%
Seizures (%)	30.0%
Ventriculoperitoneal (VP) -Shunt dependenthydrocephalus (%)	31.3%
Infections (%)	37.5%
Pneumonia (%)	17.5%
Meningitis (%)	8.8%
Others (%)	11.3%
Pneumonia + Meningitis (%)	2.5%
Pneumonia + Urinary tract infections (UTI) (%)	3.8%
Meningitis + UTI (%)	1.3%
Miscellaneous (Osteomyelitis, woundinfection) (%)	3.8%
Delayed ischemic neurological deficits (DIND) (%)	35.0%
Aneurysm location	
Anterior circulation (%)	86.3%
Posterior circulation (%)	13.8%
Glasgow outcome scale (GOS) (median)	3
1 (%)	8.8%
2 (%)	12.5%
3 (%)	30.0%
4 (%)	5.0%
5 (%)	41.3%
Modified Rankin scale (mRS) (median)	3
0 (%)	2.5%
1 (%)	31.3%
2 (%)	10.0%
3 (%)	8.8%
4 (%)	21.3%
5 (%)	17.5%
6 (%)	8.8%

## Supplementary Materials

The following are available online at www.mdpi.com/1422-0067/18/12/2580/s1.
