# Supplementary material for: Elevated Systemic IL-6 Levels in Patients with Aneurysmal Subarachnoid Hemorrhage Is an Unspecific Marker for Post-SAH Complications
Source: Int J Mol Sci. 2017 Dec 1;18(12):2580. doi: 10.3390/ijms18122580 (PMC5751183; doi:10.3390/ijms18122580)
Supplement: Supplementary file 1 [file ijms-18-02580-s001.pdf]

Supplementary Table 1: Multivariate logistic regression analysis for prediction of different complications and clinical outcome on discharge

Complications/Outcome	Days	Parameters	OR	P values
Cerebral Vasospasm (CVS)	D1	Age	0.963	0.164
		Gender	0.933	0.933
		H&H II	0.157	0.181
		H&H III	0.544	0.478
		H&H IV	1.467	0.637
		H&H V	2.587	0.325
		Fischer II	0.000	1.000
		Fischer III	0.000	0.999
		Fischer IV	1.060	0.951
		Anterior Circulation	3.428	0.167
		Clipping	1.749	0.322
		IVH	1.084	0.898
		ICB	0.639	0.513
		Log IL-6	1.083	0.891
	D3	Age	0.958	0.117
		Gender	1.067	0.913
		H&H II	0.114	0.110
		H&H III	0.702	0.685
		H&H IV	1.283	0.755
		H&H V	2.768	0.320
		Fischer II	0.000	1.000
		Fischer III	0.000	0.999
		Fischer IV	1.094	0.924
		Anterior Circulation	2.355	0.310
		Clipping	2.123	0.208
		IVH	0.642	0.502
		ICB	0.460	0.292
		Log IL-6	4.548	0.025
	D5	Age	0.952	0.085
		Gender	0.898	0.854
		H&H II	0.165	0.194
		H&H III	0.481	0.412
		H&H IV	1.718	0.508
		H&H V	2.330	0.369
		Fischer II	0.000	1.000
		Fischer III	0.000	0.999
		Fischer IV	1.108	0.913
		Anterior Circulation	1.133	0.894
		Clipping	1.339	0.619
		ICB	0.763	0.715
		IVH	0.829	0.771
		Log IL-6	3.189	0.120
	D7	Age	0.951	0.127
		Gender	1.268	0.700
		H&H II	0.127	0.141
		H&H III	0.278	0.192
		H&H IV	0.596	0.574
		H&H V	1.186	0.869
		Fischer II	0.000	1.000
		Fischer III	0.000	0.999
		Fischer IV	1.737	0.583

		Anterior Circulation	0.913	0.925
		Clipping	3.134	0.083
		ICB	0.842	0.812
		IVH	0.350	0.173
		Log IL-6	15.954	0.012
	D9	Age	0.973	0.358
		Gender	1.409	0.571
		H&H II	0.218	0.275
		H&H III	0.408	0.320
		H&H IV	0.752	0.733
		H&H V	1.254	0.826
		Fischer II	0.000	1.000
		Fischer III	0.000	0.999
		Fischer IV	1.920	0.524
		Anterior Circulation	1.889	0.458
		Clipping	3.722	0.045
		ICB	0.935	0.926
		IVH	0.327	0.146
		Log IL-6	8.578	0.014
	D11	Age	0.919	0.971
		Gender	0.892	0.847
		H&H II	0.222	0.285
		H&H III	0.363	0.293
		H&H IV	1.085	0.918
		H&H V	1.987	0.472
		Fischer II	0.000	1.000
		Fischer III	0.000	0.999
		Fischer IV	1.165	0.874
		Anterior Circulation	3.138	0.192
		Clipping	2.216	0.205
		ICB	0.606	0.494
		IVH	0.832	0.789
		Log IL-6	2.287	0.349
	D13	Age	0.963	0.194
		Gender	1.058	0.927
		H&H II	0.187	0.246
		H&H III	0.561	0.547
		H&H IV	0.841	0.837
		H&H V	1.912	0.510
		Fischer II	0.000	1.000
		Fischer III	0.000	0.999
		Fischer IV	0.783	0.810
		Anterior Circulation	3.187	0.194
		Clipping	2.517	0.145
		ICB	0.435	0.281
		IVH	0.402	0.244
		Log IL-6	5.771	0.031
**DINDs**	D1	Age	1.039	0.166
		Gender	0.443	0.174
		H&H II	0.996	0.998
		H&H III	1.536	0.647
		H&H IV	1.450	0.669
		H&H V	1.868	0.525
		Fischer II	0.000	1.000
		Fischer III	0.000	0.999
		Fischer IV	1.593	0.629

		Anterior Circulation<br>Clipping<br>ICB<br>IVH<br>Log IL-6	2.039<br>1.955<br>0.839<br>1.179<br>0.582	0.454<br>0.249<br>0.794<br>0.797<br>0.365
	D3	Age<br>Gender<br>H&H II<br>H&H III<br>H&H IV<br>H&H V<br>Fischer II<br>Fischer III<br>Fischer IV<br>Anterior Circulation<br>Clipping<br>ICB<br>IVH<br>Log IL-6	1.041<br>0.519<br>1.001<br>2.827<br>1.614<br>3.350<br>0.000<br>0.000<br>3.042<br>0.821<br>2.189<br>0.614<br>0.839<br>6.322	0.185<br>0.289<br>0.999<br>0.257<br>0.562<br>0.226<br>1.000<br>0.999<br>0.289<br>0.822<br>0.201<br>0.479<br>0.800<br>0.010
	D5	Age<br>Gender<br>H&H II<br>H&H III<br>H&H IV<br>H&H V<br>Fischer II<br>Fischer III<br>Fischer IV<br>Anterior Circulation<br>Clipping<br>ICB<br>IVH<br>Log IL-6	0.010<br>0.407<br>1.892<br>1.842<br>1.984<br>1.812<br>0.000<br>0.000<br>1.664<br>0.496<br>1.693<br>0.942<br>0.864<br>4.630	0.372<br>0.153<br>0.656<br>0.523<br>0.417<br>0.529<br>1.000<br>0.999<br>0.608<br>0.448<br>0.401<br>0.935<br>0.823<br>0.041
	D7	Age<br>Gender<br>H&H II<br>H&H III<br>H&H IV<br>H&H V<br>Fischer II<br>Fischer III<br>Fischer IV<br>Anterior Circulation<br>Clipping<br>ICB<br>IVH<br>Log IL-6	1.010<br>0.580<br>1.917<br>2.138<br>1.663<br>1.851<br>0.000<br>0.000<br>2.204<br>0.387<br>2.460<br>1.351<br>0.666<br>9.407	0.755<br>0.367<br>0.652<br>0.440<br>0.564<br>0.539<br>1.000<br>0.999<br>0.432<br>0.292<br>0.164<br>0.656<br>0.566<br>0.011
	D9	Age<br>Gender<br>H&H II<br>H&H III<br>H&H IV<br>H&H V<br>Fischer II<br>Fischer III<br>Fischer IV	1.027<br>0.611<br>4.180<br>2.872<br>1.526<br>1.676<br>0.000<br>0.000<br>2.760	0.376<br>0.428<br>0.343<br>0.286<br>0.629<br>0.607<br>1.000<br>0.999<br>0.331

		Anterior Circulation	0.517	0.452
		Clipping	3.857	0.052
		ICB	1.569	0.525
		IVH	0.449	0.295
		Log IL-6	13.118	0.005
	D11	Age	1.039	0.209
		Gender	0.330	0.099
		H&H II	2.462	0.550
		H&H III	2.442	0.404
		H&H IV	0.941	0.943
		H&H V	1.560	0.654
		Fischer II	0.000	1.000
		Fischer III	0.000	0.999
		Fischer IV	1.692	0.624
		Anterior Circulation	0.581	0.540
		Clipping	4.698	0.043
		ICB	0.735	0.669
		IVH	0.479	0.336
		Log IL-6	30.448	0.007
	D13	Age	1.037	0.216
		Gender	0.342	0.111
		H&H II	1.012	0.994
		H&H III	1.580	0.652
		H&H IV	0.769	0.766
		H&H V	1.216	0.841
		Fischer II	0.000	1.000
		Fischer III	0.000	0.999
		Fischer IV	1.056	0.959
		Anterior Circulation	0.671	0.648
		Clipping	2.820	0.131
		ICB	0.567	0.441
		IVH	0.273	0.115
		Log IL-6	7.777	0.014
**Seizures**	D1	Age	1.044	0.150
		Gender	0.618	0.470
		H&H II	0.259	0.348
		H&H III	0.087	0.027
		H&H IV	0.327	0.225
		H&H V	0.861	0.878
		Fischer II	0.000	1.000
		Fischer III	0.000	0.999
		Fischer IV	0.266	0.183
		Anterior Circulation	5.445	0.130
		Clipping	1.133	0.858
		ICB	1.156	0.850
		IVH	1.672	0.473
		Log IL-6	0.343	0.117
	D3	Age	1.024	0.419
		Gender	0.484	0.297
		H&H II	0.660	0.760
		H&H III	0.249	0.190
		H&H IV	0.660	0.623
		H&H V	1.329	0.773
		Fischer II	0.000	1.000
		Fischer III	0.000	0.999
		Fischer IV	0.227	0.144

		Anterior Circulation<br>Clipping<br>ICB<br>IVH<br>Log IL-6	3.045<br>1.475<br>1.180<br>2.015<br>2.508	0.280<br>0.561<br>0.827<br>0.342<br>0.214
	D5	Age<br>Gender<br>H&H II<br>H&H III<br>H&H IV<br>H&H V<br>Fischer II<br>Fischer III<br>Fischer IV<br>Anterior Circulation<br>Clipping<br>ICB<br>IVH<br>Log IL-6	1.029<br>0.650<br>0.603<br>0.210<br>0.628<br>1.290<br>0.000<br>0.000<br>0.320<br>3.182<br>1.329<br>1.041<br>1.450<br>1.201	0.343<br>0.509<br>0.718<br>0.148<br>0.583<br>0.778<br>1.000<br>0.999<br>0.232<br>0.309<br>0.673<br>0.958<br>0.583<br>0.821
	D7	Age<br>Gender<br>H&H II<br>H&H III<br>H&H IV<br>H&H V<br>Fischer II<br>Fischer III<br>Fischer IV<br>Anterior Circulation<br>Clipping<br>ICB<br>IVH<br>Log IL-6	1.007<br>0.586<br>0.975<br>0.198<br>0.820<br>1.618<br>0.000<br>0.000<br>0.341<br>1.816<br>1.230<br>0.676<br>1.774<br>4.221	0.836<br>0.424<br>0.985<br>0.134<br>0.817<br>0.612<br>1.000<br>0.999<br>0.274<br>0.578<br>0.771<br>0.597<br>0.418<br>0.031
	D9	Age<br>Gender<br>H&H II<br>H&H III<br>H&H IV<br>H&H V<br>Fischer II<br>Fischer III<br>Fischer IV<br>Anterior Circulation<br>Clipping<br>ICB<br>IVH<br>Log IL-6	1.018<br>0.727<br>2.290<br>0.395<br>0.783<br>1.473<br>0.000<br>0.000<br>0.459<br>2.352<br>1.772<br>0.594<br>2.164<br>9.523	0.577<br>0.639<br>0.582<br>0.389<br>0.778<br>0.686<br>1.000<br>0.999<br>0.435<br>0.412<br>0.438<br>0.492<br>0.306<br>0.020
	D11	Age<br>Gender<br>H&H II<br>H&H III<br>H&H IV<br>H&H V<br>Fischer II<br>Fischer III<br>Fischer IV	1.025<br>0.693<br>0.886<br>0.282<br>0.568<br>1.206<br>0.000<br>0.000<br>0.397	0.388<br>0.571<br>0.932<br>0.239<br>0.480<br>0.834<br>1.000<br>0.999<br>0.333

		Anterior Circulation	2.573	0.359
		Clipping	1.657	0.467
		ICB	0.825	0.783
		IVH	1.591	0.506
		Log IL-6	2.802	0.230
	D13	Age	1.022	1.022
		Gender	0.678	0.678
		H&H II	0.641	0.749
		H&H III	0.229	0.166
		H&H IV	0.423	0.306
		H&H V	0.945	0.951
		Fischer II	0.000	1.000
		Fischer III	0.000	0.999
		Fischer IV	0.398	0.345
		Anterior Circulation	2.778	0.322
		Clipping	1.625	0.477
		ICB	0.857	0.828
		IVH	1.630	0.519
		Log IL-6	2.154	0.312
**Ventriculoperitoneal Shunt dependent Chronic Hydrocephalus**	D1	Age	1.052	0.124
		Gender	0.880	0.854
		H&H II	0.223	0.326
		H&H III	0.133	0.060
		H&H IV	0.241	0.146
		H&H V	0.314	0.297
		Fischer II	0.000	1.000
		Fischer III	0.000	0.999
		Fischer IV	0.997	0.998
		Anterior Circulation	1.098	0.925
		Clipping	0.959	0.956
		ICB	2.804	0.256
		IVH	0.104	0.003
		Log IL-6	0.227	0.059
	D3	Age	1.047	0.147
		Gender	1.319	0.685
		H&H II	0.616	0.739
		H&H III	0.319	0.262
		H&H IV	0.433	0.328
		H&H V	0.939	0.953
		Fischer II	0.000	1.000
		Fischer III	0.000	1.000
		Fischer IV	1.797	0.612
		Anterior Circulation	0.407	0.359
		Clipping	1.169	0.833
		ICB	4.747	0.107
		IVH	0.076	0.002
		Log IL-6	3.381	0.092
	D5	Age	1.035	0.264
		Gender	1.284	0.702
		H&H II	0.656	0.765
		H&H III	0.322	0.244
		H&H IV	0.462	0.368
		H&H V	0.416	0.396
		Fischer II	0.000	1.000
		Fischer III	0.000	0.999
		Fischer IV	1.046	0.966

		Anterior Circulation<br>Clipping<br>ICB<br>IVH<br>Log IL-6	0.312<br>1.191<br>2.393<br>0.131<br>1.578	0.244<br>0.806<br>0.335<br>0.005<br>0.541
	D7	Age<br>Gender<br>H&H II<br>H&H III<br>H&H IV<br>H&H V<br>Fischer II<br>Fischer III<br>Fischer IV<br>Anterior Circulation<br>Clipping<br>ICB<br>IVH<br>Log IL-6	1.051<br>1.512<br>0.809<br>0.180<br>0.240<br>0.257<br>0.000<br>0.000<br>1.389<br>0.211<br>1.661<br>1.796<br>0.191<br>4.703	0.167<br>0.562<br>0.880<br>0.101<br>0.122<br>0.204<br>1.000<br>0.999<br>0.756<br>0.139<br>0.514<br>0.506<br>0.025<br>0.064
	D9	Age<br>Gender<br>H&H II<br>H&H III<br>H&H IV<br>H&H V<br>Fischer II<br>Fischer III<br>Fischer IV<br>Anterior Circulation<br>Clipping<br>ICB<br>IVH<br>Log IL-6	1.064<br>1.817<br>1.462<br>0.222<br>0.214<br>0.221<br>0.000<br>0.000<br>2.179<br>0.227<br>2.533<br>1.582<br>0.235<br>8.045	0.083<br>0.427<br>0.797<br>0.151<br>0.095<br>0.168<br>1.000<br>1.000<br>.481<br>0.157<br>0.253<br>0.601<br>0.058<br>0.025
	D11	Age<br>Gender<br>H&H II<br>H&H III<br>H&H IV<br>H&H V<br>Fischer II<br>Fischer III<br>Fischer IV<br>Anterior Circulation<br>Clipping<br>ICB<br>IVH<br>Log IL-6	1.040<br>1.389<br>1.103<br>0.467<br>0.307<br>0.390<br>0.000<br>0.000<br>0.857<br>0.277<br>1.660<br>3.296<br>0.183<br>7.031	0.191<br>0.641<br>0.949<br>0.481<br>0.173<br>0.374<br>1.000<br>0.999<br>0.883<br>0.187<br>0.502<br>0.183<br>0.022<br>0.050
	D13	Age<br>Gender<br>H&H II<br>H&H III<br>H&H IV<br>H&H V<br>Fischer II<br>Fischer III<br>Fischer IV	1.035<br>1.375<br>0.424<br>0.415<br>0.163<br>0.254<br>0.000<br>0.000<br>0.717	0.263<br>0.660<br>0.611<br>0.415<br>0.053<br>0.200<br>1.000<br>0.999<br>0.764

		Anterior Circulation	0.234	0.157
		Clipping	1.756	0.460
		ICB	3.615	0.162
		IVH	0.297	0.127
		Log IL-6	9.571	0.025
**Cerebral Ischemia/ Cerebral Infarction**	D1	Age	0.996	0.899
		Gender	0.564	0.339
		H&H II	1.975	0.586
		H&H III	0.549	0.520
		H&H IV	1.015	0.986
		H&H V	0.846	0.869
		Fischer II	1828620772	1.000
		Fischer III	0.000	0.999
		Fischer IV	0.172	0.094
		Anterior Circulation	2.186	0.384
		Clipping	1.430	0.558
		ICB	0.887	0.861
		IVH	0.486	0.273
		Log IL-6	0.221	0.024
	D3	Age	0.997	0.893
		Gender	0.736	0.591
		H&H II	3.552	0.277
		H&H III	0.690	0.663
		H&H IV	1.276	0.745
		H&H V	1.215	0.834
		Fischer II	1735454054	1.000
		Fischer III	0.000	0.999
		Fischer IV	0.349	0.269
		Anterior Circulation	0.817	0.803
		Clipping	1.321	0.634
		ICB	0.960	0.952
		IVH	0.523	0.305
		Log IL-6	1.565	0.455
	D5	Age	0.993	0.785
		Gender	0.739	0.594
		H&H II	3.940	0.257
		H&H III	0.759	0.754
		H&H IV	1.373	0.686
		H&H V	0.868	0.876
		Fischer II	996363628	1.000
		Fischer III	0.000	0.999
		Fischer IV	0.239	0.135
		Anterior Circulation	0.581	0.545
		Clipping	1.549	0.463
		ICB	1.072	0.922
		IVH	0.616	0.434
		Log IL-6	1.693	0.439
	D7	Age	1.003	0.924
		Gender	0.839	0.751
		H&H II	3.552	0.281
		H&H III	0.589	0.521
		H&H IV	0.861	0.850
		H&H V	0.648	0.638
		Fischer II	1588347521	1.000
		Fischer III	0.000	0.999
		Fischer IV	0.300	0.208

		Anterior Circulation	0.576	0.518
		Clipping	1.858	0.314
		ICB	0.863	0.823
		IVH	0.768	0.681
		Log IL-6	2.027	0.224
	D9	Age	1.013	0.664
		Gender	0.875	0.818
		H&H II	5.794	0.156
		H&H III	0.632	0.607
		H&H IV	0.719	0.678
		H&H V	0.529	0.497
		Fischer II	677806370	1.000
		Fischer III	0.000	0.999
		Fischer IV	0.323	0.256
		Anterior Circulation	0.653	0.606
		Clipping	2.723	0.120
		ICB	0.757	0.680
		IVH	1.160	0.830
		Log IL-6	3.877	0.081
	D11	Age	1.014	0.606
		Gender	0.865	0.811
		H&H II	17.451	0.045
		H&H III	1.309	0.791
		H&H IV	1.280	0.757
		H&H V	1.092	0.925
		Fischer II	252495894.300	1.000
		Fischer III	0.000	0.999
		Fischer IV	0.168	0.069
		Anterior Circulation	1.186	0.852
		Clipping	2.829	0.121
		ICB	1.800	0.411
		IVH	0.898	0.876
		Log IL-6	4.974	0.089
	D13	Age	1.013	0.645
		Gender	0.898	0.862
		H&H II	14.898	0.060
		H&H III	1.313	0.785
		H&H IV	1.218	0.816
		H&H V	1.044	0.964
		Fischer II	198985840	1.000
		Fischer III	0.000	0.999
		Fischer IV	0.108	0.031
		Anterior Circulation	1.291	0.787
		Clipping	2.454	0.170
		ICB	2.415	0.237
		IVH	1.491	0.604
		Log IL-6	3.577	0.087
**Interventional Cerebral Ischemia**	D1	Age	1.001	0.970
		Gender	0.905	0.894
		H&H II	5.170	0.267
		H&H III	0.669	0.723
		H&H IV	1.109	0.916
		H&H V	0.465	0.541
		Fischer II	0.000	1.000
		Fischer III	0.000	0.999
		Fischer IV	0.273	0.228

		Anterior Circulation<br>Clipping<br>ICB<br>IVH<br>Log IL-6	860563744.400<br>0.969<br>1.065<br>0.263<br>0.718	0.999<br>0.968<br>0.942<br>0.068<br>0.638
	D3	Age<br>Gender<br>H&H II<br>H&H III<br>H&H IV<br>H&H V<br>Fischer II<br>Fischer III<br>Fischer IV<br>Anterior Circulation<br>Clipping<br>ICB<br>IVH<br>Log IL-6	1.011<br>1.425<br>2.304<br>0.213<br>0.656<br>0.217<br>0.000<br>0.000<br>0.458<br>3.802<br>0.572<br>0.711<br>0.261<br>0.521	0.740<br>0.620<br>0.517<br>0.154<br>0.632<br>0.221<br>1.000<br>0.999<br>0.456<br>0.279<br>0.465<br>0.691<br>0.065<br>0.373
	D5	Age<br>Gender<br>H&H II<br>H&H III<br>H&H IV<br>H&H V<br>Fischer II<br>Fischer III<br>Fischer IV<br>Anterior Circulation<br>Clipping<br>ICB<br>IVH<br>Log IL-6	1.011<br>1.710<br>1.786<br>0.332<br>0.577<br>0.197<br>0.000<br>0.000<br>0.416<br>5.209<br>0.926<br>1.066<br>0.320<br>0.491	0.727<br>0.430<br>0.652<br>0.292<br>0.533<br>0.165<br>1.000<br>0.999<br>0.393<br>0.218<br>0.913<br>0.940<br>0.112<br>0.405
	D7	Age<br>Gender<br>H&H II<br>H&H III<br>H&H IV<br>H&H V<br>Fischer II<br>Fischer III<br>Fischer IV<br>Anterior Circulation<br>Clipping<br>ICB<br>IVH<br>Log IL-6	1.019<br>1.512<br>1.990<br>0.341<br>0.639<br>0.208<br>0.000<br>0.000<br>0.404<br>4.857<br>0.842<br>1.032<br>0.316<br>0.485	0.585<br>0.546<br>0.587<br>0.295<br>0.628<br>0.182<br>1.000<br>0.999<br>0.379<br>0.209<br>0.815<br>0.969<br>0.124<br>0.385
	D9	Age<br>Gender<br>H&H II<br>H&H III<br>H&H IV<br>H&H V<br>Fischer II<br>Fischer III<br>Fischer IV	1.015<br>1.609<br>2.047<br>0.352<br>0.572<br>0.190<br>0.000<br>0.000<br>0.401	0.664<br>0.498<br>0.581<br>0.307<br>0.533<br>0.158<br>1.000<br>0.999<br>0.387

		Anterior Circulation<br>Clipping<br>ICB<br>IVH<br>Log IL-6	4.375<br>0.828<br>1.051<br>0.297<br>0.705	0.228<br>0.798<br>0.951<br>0.116<br>0.687
	D11	Age<br>Gender<br>H&H II<br>H&H III<br>H&H IV<br>H&H V<br>Fischer II<br>Fischer III<br>Fischer IV<br>Anterior Circulation<br>Clipping<br>ICB<br>IVH<br>Log IL-6	1.000<br>2.492<br>6.141<br>0.651<br>0.670<br>0.201<br>0.000<br>0.000<br>0.341<br>4.267<br>0.857<br>1.268<br>0.355<br>1.835	0.998<br>0.206<br>0.197<br>0.691<br>0.653<br>0.177<br>1.000<br>0.999<br>0.300<br>0.246<br>0.837<br>0.778<br>0.168<br>0.512
	D13	Age<br>Gender<br>H&H II<br>H&H III<br>H&H IV<br>H&H V<br>Fischer II<br>Fischer III<br>Fischer IV<br>Anterior Circulation<br>Clipping<br>ICB<br>IVH<br>Log IL-6	0.994<br>2.869<br>7.240<br>0.925<br>0.719<br>0.215<br>0.000<br>0.000<br>0.200<br>5.307<br>0.805<br>1.860<br>0.567<br>2.562	0.857<br>0.158<br>0.175<br>0.944<br>0.740<br>0.210<br>1.000<br>0.999<br>0.149<br>0.206<br>0.778<br>0.494<br>0.501<br>0.262
**Delayed Cerebral Ischemia (DCI)**	D1	Age<br>Gender<br>H&H II<br>H&H III<br>H&H IV<br>H&H V<br>Fischer II<br>Fischer III<br>Fischer IV<br>Anterior Circulation<br>Clipping<br>ICB<br>IVH<br>Log IL-6	1.005<br>0.469<br>0.669<br>0.654<br>1.018<br>1.771<br>5442967125<br>0.000<br>0.248<br>0.472<br>2.437<br>1.159<br>2.303<br>0.145	0.890<br>0.307<br>0.806<br>0.737<br>0.988<br>0.654<br>1.000<br>0.999<br>0.263<br>0.452<br>0.288<br>0.871<br>0.429<br>0.026
	D3	Age<br>Gender<br>H&H II<br>H&H III<br>H&H IV<br>H&H V<br>Fischer II<br>Fischer III<br>Fischer IV	0.977<br>0.342<br>1.738<br>4.629<br>3.166<br>9.362<br>2359841321<br>0.000<br>0.378	0.467<br>0.193<br>0.756<br>0.224<br>0.272<br>0.074<br>1.000<br>0.999<br>0.391

		Anterior Circulation<br>Clipping<br>ICB<br>IVH<br>Log IL-6	0.188<br>2.913<br>1.245<br>1.927<br>6.608	0.104<br>0.153<br>0.800<br>0.454<br>0.040
	D5	Age<br>Gender<br>H&H II<br>H&H III<br>H&H IV<br>H&H V<br>Fischer II<br>Fischer III<br>Fischer IV<br>Anterior Circulation<br>Clipping<br>ICB<br>IVH<br>Log IL-6	0.974<br>0.344<br>5.180<br>2.571<br>4.070<br>5.239<br>4034782387<br>0.000<br>0.288<br>0.087<br>2.741<br>1.023<br>1.859<br>6.386	0.460<br>0.200<br>0.325<br>0.475<br>0.216<br>0.171<br>1.000<br>0.999<br>0.289<br>0.038<br>0.241<br>0.981<br>0.482<br>0.056
	D7	Age<br>Gender<br>H&H II<br>H&H III<br>H&H IV<br>H&H V<br>Fischer II<br>Fischer III<br>Fischer IV<br>Anterior Circulation<br>Clipping<br>ICB<br>IVH<br>Log IL-6	0.973<br>0.518<br>4.804<br>2.747<br>2.769<br>4.907<br>23469460290<br>0.000<br>0.371<br>0.058<br>4.541<br>0.618<br>3.212<br>8.273	0.463<br>0.386<br>0.348<br>0.427<br>0.389<br>0.218<br>1.000<br>0.999<br>0.405<br>0.019<br>0.101<br>0.589<br>0.213<br>0.008
	D9	Age<br>Gender<br>H&H II<br>H&H III<br>H&H IV<br>H&H V<br>Fischer II<br>Fischer III<br>Fischer IV<br>Anterior Circulation<br>Clipping<br>ICB<br>IVH<br>Log IL-6	1.001<br>0.442<br>30.807<br>5.414<br>1.885<br>4.434<br>2001809672<br>0.000<br>0.395<br>0.041<br>21.735<br>0.436<br>9.224<br>30.388	0.972<br>0.349<br>0.096<br>0.298<br>0.604<br>0.261<br>1.000<br>0.999<br>0.512<br>0.017<br>0.012<br>0.424<br>0.080<br>0.010
	D11	Age<br>Gender<br>H&H II<br>H&H III<br>H&H IV<br>H&H V<br>Fischer II<br>Fischer III<br>Fischer IV	1.025<br>0.326<br>17.805<br>3.104<br>3.272<br>8.022<br>914552763.600<br>0.000<br>0.164	0.472<br>0.255<br>0.148<br>0.541<br>0.369<br>0.142<br>1.000<br>0.999<br>0.191

		Anterior Circulation<br>Clipping<br>ICB<br>IVH<br>Log IL-6	0.191<br>11.607<br>1.954<br>4.548<br>7.405	0.183<br>0.033<br>0.518<br>0.188<br>0.102
	D13	Age<br>Gender<br>H&H II<br>H&H III<br>H&H IV<br>H&H V<br>Fischer II<br>Fischer III<br>Fischer IV<br>Anterior Circulation<br>Clipping<br>ICB<br>IVH<br>Log IL-6	1.029<br>0.262<br>10.003<br>2.032<br>2.037<br>5.541<br>748691625.000<br>0.000<br>0.101<br>0.242<br>12.048<br>2.971<br>8.253<br>4.754	0.428<br>0.177<br>0.250<br>0.686<br>0.583<br>0.216<br>1.000<br>0.999<br>0.109<br>0.258<br>0.040<br>0.297<br>0.110<br>0.112
**Infections**	D1	Age<br>Gender<br>H&H II<br>H&H III<br>H&H IV<br>H&H V<br>Fischer II<br>Fischer III<br>Fischer IV<br>Anterior Circulation<br>Clipping<br>ICB<br>IVH<br>Log IL-6	0.975<br>14.193<br>0.000<br>0.019<br>0.630<br>0.667<br>0.000<br>0.000<br>1.238<br>9.609<br>0.317<br>1.070<br>0.871<br>0.513	0.440<br>0.005<br>0.999<br>0.002<br>0.608<br>0.714<br>1.000<br>0.999<br>0.837<br>0.079<br>0.135<br>0.930<br>0.848<br>0.365
	D3	Age<br>Gender<br>H&H II<br>H&H III<br>H&H IV<br>H&H V<br>Fischer II<br>Fischer III<br>Fischer IV<br>Anterior Circulation<br>Clipping<br>ICB<br>IVH<br>Log IL-6	0.967<br>15.902<br>0.000<br>0.026<br>0.776<br>1.011<br>0.000<br>0.000<br>1.485<br>5.624<br>0.378<br>1.204<br>1.152<br>2.862	0.277<br>0.004<br>0.999<br>0.005<br>0.765<br>0.992<br>1.000<br>1.000<br>0.708<br>0.159<br>0.209<br>0.817<br>0.848<br>0.192
	D5	Age<br>Gender<br>H&H II<br>H&H III<br>H&H IV<br>H&H V<br>Fischer II<br>Fischer III<br>Fischer IV	0.971<br>28.631<br>0.000<br>0.010<br>0.514<br>0.644<br>0.000<br>0.000<br>1.378	0.403<br>0.003<br>0.999<br>0.003<br>0.481<br>0.687<br>1.000<br>1.000<br>0.763

		Anterior Circulation<br>Clipping<br>ICB<br>IVH<br>Log IL-6	19.101<br>0.319<br>0.895<br>1.020<br>1.898	0.081<br>0.173<br>0.903<br>0.980<br>0.469
	D7	Age<br>Gender<br>H&H II<br>H&H III<br>H&H IV<br>H&H V<br>Fischer II<br>Fischer III<br>Fischer IV<br>Anterior Circulation<br>Clipping<br>ICB<br>IVH<br>Log IL-6	0.956<br>35.873<br>0.000<br>0.012<br>0.474<br>0.800<br>0.000<br>0.000<br>1.200<br>18.541<br>0.319<br>1.237<br>0.842<br>3.617	0.239<br>0.002<br>0.999<br>0.003<br>0.443<br>0.852<br>1.000<br>1.000<br>0.871<br>0.081<br>0.216<br>0.802<br>0.828<br>0.138
	D9	Age<br>Gender<br>H&H II<br>H&H III<br>H&H IV<br>H&H V<br>Fischer II<br>Fischer III<br>Fischer IV<br>Anterior Circulation<br>Clipping<br>ICB<br>IVH<br>Log IL-6	0.942<br>26.186<br>0.000<br>0.026<br>0.831<br>1.035<br>0.000<br>0.000<br>1.787<br>6.476<br>0.292<br>0.912<br>1.073<br>4.855	0.114<br>0.002<br>0.999<br>0.007<br>0.837<br>0.977<br>1.000<br>1.000<br>0.625<br>0.157<br>0.184<br>0.912<br>0.929<br>0.089
	D11	Age<br>Gender<br>H&H II<br>H&H III<br>H&H IV<br>H&H V<br>Fischer II<br>Fischer III<br>Fischer IV<br>Anterior Circulation<br>Clipping<br>ICB<br>IVH<br>Log IL-6	0.927<br>47.449<br>0.000<br>0.011<br>0.318<br>0.842<br>0.000<br>0.000<br>1.454<br>2.492<br>0.346<br>0.793<br>4.387<br>116.017	0.072<br>0.003<br>0.999<br>0.014<br>0.299<br>0.900<br>1.000<br>1.000<br>0.781<br>0.573<br>0.265<br>0.806<br>0.139<br>0.006
	D13	Age<br>Gender<br>H&H II<br>H&H III<br>H&H IV<br>H&H V<br>Fischer II<br>Fischer III<br>Fischer IV	0.939<br>27.468<br>0.000<br>0.012<br>0.223<br>0.602<br>0.000<br>0.000<br>1.008	0.098<br>0.003<br>0.999<br>0.007<br>0.161<br>0.677<br>1.000<br>1.000<br>0.995

		Anterior Circulation	3.811	0.389
		Clipping	0.261	0.135
		ICB	1.152	0.868
		IVH	4.831	0.133
		Log IL-6	11.896	0.034
**Pneumonia**	D1	Age	0.949	0.170
		Gender	4.194	0.140
		H&H II	0.000	0.999
		H&H III	0.044	0.040
		H&H IV	1.506	0.685
		H&H V	0.056	0.080
		Fischer II	0.000	1.000
		Fischer III	0.000	1.000
		Fischer IV	0.997	0.998
		Anterior Circulation	1.173	0.892
		Clipping	0.141	0.057
		ICB	1.071	0.952
		IVH	0.079	0.011
		Log IL-6	0.677	0.650
	D3	Age	0.941	0.112
		Gender	5.665	0.070
		H&H II	0.000	0.999
		H&H III	0.022	0.044
		H&H IV	1.553	0.651
		H&H V	0.000	0.998
		Fischer II	0.000	1.000
		Fischer III	0.000	1.000
		Fischer IV	0.734	0.824
		Anterior Circulation	0.436	0.472
		Clipping	0.173	0.096
		ICB	1.251	0.857
		IVH	0.111	0.044
		Log IL-6	5.396	0.084
	D5	Age	0.969	0.407
		Gender	8.879	0.028
		H&H II	0.000	0.999
		H&H III	0.027	0.033
		H&H IV	0.819	0.841
		H&H V	0.023	0.023
		Fischer II	0.000	1.000
		Fischer III	0.000	1.000
		Fischer IV	1.232	0.866
		Anterior Circulation	0.982	0.989
		Clipping	0.234	0.133
		ICB	1.056	0.963
		IVH	0.095	0.018
		Log IL-6	1.939	0.479
	D7	Age	0.963	0.356
		Gender	12.027	0.021
		H&H II	0.000	0.999
		H&H III	0.004	0.043
		H&H IV	0.578	0.585
		H&H V	0.015	0.018
		Fischer II	0.000	1.000
		Fischer III	0.000	1.000
		Fischer IV	1.282	0.848

		Anterior Circulation	0.748	0.827
		Clipping	0.245	0.158
		ICB	0.992	0.994
		IVH	0.082	0.023
		Log IL-6	5.185	0.103
	D9	Age	0.936	0.138
		Gender	24.316	0.018
		H&H II	0.000	0.999
		H&H III	0.000	0.998
		H&H IV	1.010	0.992
		H&H V	0.008	0.014
		Fischer II	61.996	1.000
		Fischer III	29.054	1.000
		Fischer IV	5.187	0.310
		Anterior Circulation	0.387	0.451
		Clipping	0.115	0.076
		ICB	0.388	0.454
		IVH	0.108	0.050
		Log IL-6	31.078	0.019
	D11	Age	0.913	0.120
		Gender	101.013	0.056
		H&H II	0.000	0.999
		H&H III	0.000	0.998
		H&H IV	0.471	0.685
		H&H V	0.005	0.064
		Fischer II	8.417	1.000
		Fischer III	202.041	1.000
		Fischer IV	1.790	0.739
		Anterior Circulation	0.064	0.218
		Clipping	0.139	0.213
		ICB	1.009	0.996
		IVH	0.145	0.152
		Log IL-6	1548.803	0.038
	D13	Age	0.892	0.075
		Gender	28.507	0.032
		H&H II	0.000	0.999
		H&H III	0.000	0.998
		H&H IV	0.774	0.853
		H&H V	0.008	0.023
		Fischer II	9.684	1.000
		Fischer III	10.052	1.000
		Fischer IV	0.446	0.622
		Anterior Circulation	0.271	0.401
		Clipping	0.074	0.084
		ICB	2.722	0.481
		IVH	0.254	0.256
		Log IL-6	45.363	0.016
**Meningitis**	D1	Age	0.972	0.672
		Gender	5.967	0.089
		H&H II	0.025	1.000
		H&H III	10241781.340	0.998
		H&H IV	24143971.57	0.998
		H&H V	95670650.6	0.998
		Fischer II	0.094	1.000
		Fischer III	0.030	1.000
		Fischer IV	9977268.341	0.999

		Anterior Circulation<br>Clipping<br>ICB<br>IVH<br>Log IL-6	51712516.920<br>0.752<br>3.175<br>123917324.600<br>0.162	0.999<br>0.816<br>0.445<br>0.998<br>0.225
	D3	Age<br>Gender<br>H&H II<br>H&H III<br>H&H IV<br>H&H V<br>Fischer II<br>Fischer III<br>Fischer IV<br>Anterior Circulation<br>Clipping<br>ICB<br>IVH<br>Log IL-6	1.079<br>59.621<br>0.001<br>101631.438<br>4935355.211<br>18228025.170<br>0.032<br>0.012<br>2744592.516<br>85061044.300<br>23.050<br>6.942<br>177797142.300<br>0.002	0.354<br>0.026<br>1.000<br>0.999<br>0.998<br>0.998<br>1.000<br>1.000<br>0.998<br>0.998<br>0.139<br>0.297<br>0.997<br>0.024
	D5	Age<br>Gender<br>H&H II<br>H&H III<br>H&H IV<br>H&H V<br>Fischer II<br>Fischer III<br>Fischer IV<br>Anterior Circulation<br>Clipping<br>ICB<br>IVH<br>Log IL-6	0.982<br>4.574<br>0.052<br>9346048.122<br>65956150.370<br>142729031.900<br>1.361<br>0.448<br>50274126.530<br>34513696.840<br>0.520<br>1.399<br>79128992.730<br>0.180	0.825<br>0.196<br>1.000<br>0.998<br>0.998<br>0.998<br>1.000<br>1.000<br>0.999<br>0.999<br>0.646<br>0.845<br>0.998<br>0.321
	D7	Age<br>Gender<br>H&H II<br>H&H III<br>H&H IV<br>H&H V<br>Fischer II<br>Fischer III<br>Fischer IV<br>Anterior Circulation<br>Clipping<br>ICB<br>IVH<br>Log IL-6	1.041<br>7.162<br>0.079<br>16065571.910<br>126341346.400<br>184247988.200<br>0.020<br>1.286<br>48867519.290<br>39480808.620<br>1.107<br>2.081<br>92917410.950<br>0.080	0.594<br>0.102<br>1.000<br>0.998<br>0.998<br>0.998<br>1.000<br>1.000<br>0.999<br>0.999<br>0.938<br>0.608<br>0.998<br>0.207
	D9	Age<br>Gender<br>H&H II<br>H&H III<br>H&H IV<br>H&H V<br>Fischer II<br>Fischer III<br>Fischer IV	1.016<br>5.200<br>0.088<br>15562415.290<br>85632280.530<br>159265770.400<br>0.212<br>1.157<br>70568951.840	0.814<br>0.136<br>1.000<br>0.998<br>0.998<br>0.998<br>1.000<br>1.000<br>0.999

		Anterior Circulation	45403637.070	0.999
		Clipping	0.946	0.964
		ICB	2.117	0.595
		IVH	79790351.480	0.998
		Log IL-6	0.215	0.249
	D11	Age	0.948	0.480
		Gender	3.066	0.323
		H&H II	0.090	1.000
		H&H III	7576173.303	0.999
		H&H IV	65150128.340	0.998
		H&H V	126199614.600	0.998
		Fischer II	2.194	1.000
		Fischer III	0.203	1.000
		Fischer IV	35742593.970	0.999
		Anterior Circulation	96885011.000	0.999
		Clipping	0.334	0.417
		ICB	1.063	0.969
		IVH	52634065.220	0.998
		Log IL-6	0.150	0.303
	D13	Age	0.967	0.664
		Gender	4.555	0.205
		H&H II	0.091	1.000
		H&H III	6423895.200	0.999
		H&H IV	68784426.510	0.999
		H&H V	194022657.100	0.998
		Fischer II	0.970	1.000
		Fischer III	0.369	1.000
		Fischer IV	47603910.710	0.999
		Anterior Circulation	77588897.800	0.999
		Clipping	0.528	0.640
		ICB	0.860	0.927
		IVH	52002653.210	0.998
		Log IL-6	0.079	0.103
**Others**	D1	Age	1.119	0.124
		Gender	47.049	0.032
		H&H II	0.000	0.999
		H&H III	0.000	0.997
		H&H IV	0.007	0.063
		H&H V	0.643	0.776
		Fischer II	0.001	1.000
		Fischer III	2.267	1.000
		Fischer IV	0.041	0.147
		Anterior Circulation	2758776526.000	0.998
		Clipping	10.329	0.196
		ICB	3.802	0.453
		IVH	24.882	0.097
		Log IL-6	0.405	0.540
	D3	Age	1.056	0.590
		Gender	28.694	0.068
		H&H II	0.000	0.999
		H&H III	0.000	0.997
		H&H IV	0.006	0.116
		H&H V	41.110	0.223
		Fischer II	0.002	1.000
		Fischer III	152.089	1.000
		Fischer IV	0.027	0.246

		Anterior Circulation	5341252110.000	0.998
		Clipping	4.453	0.431
		ICB	4.908	0.487
		IVH	14.619	0.311
		Log IL-6	677.687	0.145
	D5	Age	1.061	0.442
		Gender	31.585	0.035
		H&H II	0.000	0.999
		H&H III	0.000	0.998
		H&H IV	0.018	0.080
		H&H V	1.268	0.860
		Fischer II	0.000	1.000
		Fischer III	2.950	1.000
		Fischer IV	0.041	0.151
		Anterior Circulation	720489413.100	0.998
		Clipping	9.897	0.184
		ICB	1.682	0.782
		IVH	20.393	0.114
		Log IL-6	10.272	0.365
	D7	Age	1.035	0.864
		Gender	187.204	0.138
		H&H II	0.000	0.999
		H&H III	0.000	0.996
		H&H IV	0.006	0.306
		H&H V	4.133	0.604
		Fischer II	105056.134	1.000
		Fischer III	465129.150	1.000
		Fischer IV	0.341	0.763
		Anterior Circulation	902834446.200	0.998
		Clipping	1.500	0.884
		ICB	0.254	0.679
		IVH	26.913	0.313
		Log IL-6	1557.556	0.076
	D9	Age	1.090	0.502
		Gender	5582.151	0.064
		H&H II	0.000	0.999
		H&H III	0.000	0.997
		H&H IV	0.000	0.101
		H&H V	0.372	0.664
		Fischer II	0.000	1.000
		Fischer III	64.699	1.000
		Fischer IV	0.000	0.230
		Anterior Circulation	24459366810.000	0.998
		Clipping	349.070	0.133
		ICB	5.217	0.718
		IVH	6685.641	0.153
		Log IL-6	1206.305	0.078
	D11	Age	1.167	0.240
		Gender	171.657	0.041
		H&H II	0.000	0.999
		H&H III	0.000	0.998
		H&H IV	0.002	0.075
		H&H V	1.649	0.771
		Fischer II	0.000	1.000
		Fischer III	208.026	1.000
		Fischer IV	0.008	0.197

		Anterior Circulation	8249217656.000	0.998
		Clipping	188.567	0.142
		ICB	8.962	0.447
		IVH	69.311	0.120
		Log IL-6	268.876	0.063
	D13	Age	1.169	0.171
		Gender	98.175	0.058
		H&H II	0.000	0.999
		H&H III	0.000	0.997
		H&H IV	0.000	0.055
		H&H V	1.222	0.911
		Fischer II	0.000	1.000
		Fischer III	369.442	1.000
		Fischer IV	0.001	0.177
		Anterior Circulation	284479100100	0.998
		Clipping	216.610	0.115
		ICB	91.344	0.351
		IVH	301.049	0.077
		Log IL-6	6717.831	0.095
**Clinical Outcome Glasgow Outcome Scale (GOS 1 – 3 = Poor Outcome; GOS 4 – 5 = Good Outcome)**	D1	Age	0.992	0.828
		Gender	1.860	0.424
		H&H II	24.331	0.045
		H&H III	30.540	0.008
		H&H IV	12.069	0.044
		H&H V	1.563	0.730
		Fischer II	0.009	1.000
		Fischer III	4.256 x 10^{17}	0.999
		Fischer IV	366730862.6	0.999
		Anterior Circulation	0.163	0.133
		Clipping	3.863	0.107
		ICB	2.703	0.266
		IVH	12.207	0.018
		Log IL-6	2.201	0.383
	D3	Age	0.983	0.658
		Gender	1.367	0.671
		H&H II	14.342	0.074
		H&H III	13.524	0.040
		H&H IV	6.692	0.102
		H&H V	1.430	0.793
		Fischer II	0.026	1.000
		Fischer III	1.658 x 10^{17}	0.999
		Fischer IV	186617455.300	0.999
		Anterior Circulation	0.251	0.206
		Clipping	4.726	0.080
		ICB	2.531	0.300
		IVH	6.327	0.097
		Log IL-6	0.204	0.097
	D5	Age	0.998	0.965
		Gender	1.201	0.811
		H&H II	9.447	0.149
		H&H III	12.076	0.046
		H&H IV	5.603	0.139
		H&H V	1.620	0.714
		Fischer II	0.018	1.000
		Fischer III	1.969 x 10^{17}	0.999
		Fischer IV	191288043.6	0.999

		Anterior Circulation	0.709	0.770
		Clipping	4.000	0.104
		ICB	3.769	0.143
		IVH	5.854	0.095
		Log IL-6	0.238	0.151
	D7	Age	1.001	0.972
		Gender	1.121	0.882
		H&H II	7.861	0.172
		H&H III	15.749	0.026
		H&H IV	6.526	0.102
		H&H V	1.204	0.889
		Fischer II	0.002	1.000
		Fischer III	1.365×10^{17}	0.999
		Fischer IV	178277944.4	0.999
		Anterior Circulation	0.772	0.823
		Clipping	4.174	0.114
		ICB	3.427	0.171
		IVH	5.750	0.125
		Log IL-6	0.193	0.100
	D9	Age	0.988	0.758
		Gender	0.996	0.996
		H&H II	4.469	0.352
		H&H III	12.665	0.048
		H&H IV	7.862	0.069
		H&H V	1.522	0.758
		Fischer II	0.013	1.000
		Fischer III	2.025×10^{17}	0.999
		Fischer IV	194286562.5	0.999
		Anterior Circulation	0.512	0.555
		Clipping	3.450	0.167
		ICB	3.979	0.131
		IVH	3.455	0.278
		Log IL-6	0.077	0.032
	D11	Age	0.956	0.295
		Gender	1.027	0.975
		H&H II	2.180	0.633
		H&H III	5.124	0.215
		H&H IV	6.187	0.113
		H&H V	0.984	0.990
		Fischer II	0.060	1.000
		Fischer III	7.126×10^{16}	0.999
		Fischer IV	174545282.500	0.999
		Anterior Circulation	0.291	0.300
		Clipping	1.866	0.507
		ICB	2.307	0.351
		IVH	4.406	0.175
		Log IL-6	0.065	0.030
	D13	Age	0.965	0.407
		Gender	1.010	0.990
		H&H II	4.572	0.341
		H&H III	4.979	0.213
		H&H IV	10.425	0.049
		H&H V	1.440	0.783
		Fischer II	0.066	1.000
		Fischer III	1.058×10^{17}	0.999
		Fischer IV	277445895.3	0.999

		Anterior Circulation	0.323	0.346
		Clipping	1.766	0.535
		ICB	1.671	0.559
		IVH	1.837	0.602
		Log IL-6	0.053	0.020
**Clinical Outcome Modified Rankin Scale (mRS 0 – 2 = Good Outcome; mRS 3 – 5 = Poor Outcome)**	D1	Age	1.030	0.518
		Gender	0.431	0.316
		H&H II	0.000	0.998
		H&H III	0.000	0.998
		H&H IV	0.000	0.998
		H&H V	0.000	0.998
		Fischer II	11.858	1.000
		Fischer III	0.000	0.999
		Fischer IV	0.000	0.998
		Anterior Circulation	2.920	0.438
		Clipping	1.063	0.947
		ICB	0.814	0.833
		IVH	0.030	0.009
		Log IL-6	0.118	0.059
	D3	Age	1.055	0.282
		Gender	0.334	0.242
		H&H II	0.000	0.998
		H&H III	0.000	0.998
		H&H IV	0.000	0.998
		H&H V	0.000	0.998
		Fischer II	4.490	1.000
		Fischer III	0.000	0.999
		Fischer IV	0.000	0.999
		Anterior Circulation	2.220	0.598
		Clipping	0.958	0.964
		ICB	0.825	0.847
		IVH	0.142	0.150
		Log IL-6	18.325	0.019
	D5	Age	1.025	0.583
		Gender	0.533	0.445
		H&H II	0.000	0.998
		H&H III	0.000	0.998
		H&H IV	0.000	0.998
		H&H V	0.000	0.998
		Fischer II	18.186	1.000
		Fischer III	0.000	0.999
		Fischer IV	0.000	0.999
		Anterior Circulation	0.720	0.808
		Clipping	0.997	0.997
		ICB	0.502	0.470
		IVH	0.103	0.069
		Log IL-6	3.017	0.275
	D7	Age	1.001	0.990
		Gender	0.446	0.402
		H&H II	0.000	0.998
		H&H III	0.000	0.998
		H&H IV	0.000	0.998
		H&H V	0.000	0.998
		Fischer II	408.022	1.000
		Fischer III	0.000	0.999
		Fischer IV	0.000	0.999

		Anterior Circulation	0.258	0.343
		Clipping	1.321	0.780
		ICB	0.285	0.205
		IVH	0.323	0.404
		Log IL-6	103.556	0.016
	D9	Age	1.040	0.422
		Gender	0.434	0.414
		H&H II	0.000	0.998
		H&H III	0.000	0.998
		H&H IV	0.000	0.998
		H&H V	0.000	0.998
		Fischer II	13.549	1.000
		Fischer III	0.000	0.999
		Fischer IV	0.000	0.999
		Anterior Circulation	0.504	0.627
		Clipping	2.233	0.434
		ICB	0.127	0.066
		IVH	1.179	0.914
		Log IL-6	311.201	0.007
	D11	Age	1.063	0.192
		Gender	0.566	0.540
		H&H II	0.000	0.998
		H&H III	0.000	0.998
		H&H IV	0.000	0.998
		H&H V	0.000	0.998
		Fischer II	3.522	1.000
		Fischer III	0.000	0.999
		Fischer IV	0.000	0.999
		Anterior Circulation	0.821	0.878
		Clipping	3.369	0.269
		ICB	0.576	0.566
		IVH	0.287	0.363
		Log IL-6	66.778	0.027
	D13	Age	1.072	0.200
		Gender	0.382	0.340
		H&H II	0.000	0.998
		H&H III	0.000	0.998
		H&H IV	0.000	0.998
		H&H V	0.000	0.998
		Fischer II	3.703	1.000
		Fischer III	0.000	0.999
		Fischer IV	0.000	0.999
		Anterior Circulation	1.011	0.994
		Clipping	3.531	0.251
		ICB	0.692	0.699
		IVH	0.591	0.701
		Log IL-6	48.416	0.006
